# Supplementary material for: Disruption of the PIKfyve complex unveils an adaptive mechanism to promote lysosomal repair and mitochondrial homeostasis
Source: Nat Commun. 2025 Nov 28;16:10761. doi: 10.1038/s41467-025-65798-6 (PMC12663346; doi:10.1038/s41467-025-65798-6)
Supplement: Supplementary file 1 — Supplementary Information [file 41467_2025_65798_MOESM1_ESM.pdf]

Supplementary Information

Disruption of the PIKfyve complex unveils an adaptive mechanism to promote lysosomal repair and mitochondrial homeostasis

Candice Kutchukian[1], Maria Casas Prat[1], Rose Ellen Dixon[1], and Eamonn James Dickson[1*]

Affiliations:
[1]Department of Physiology and Membrane Biology, University of California, Davis, California, 95616.

*Corresponding author:
Eamonn Dickson
Email: ejdickson@ucdavis.edu
Phone: (530) 752-6195

Supplementary Figures

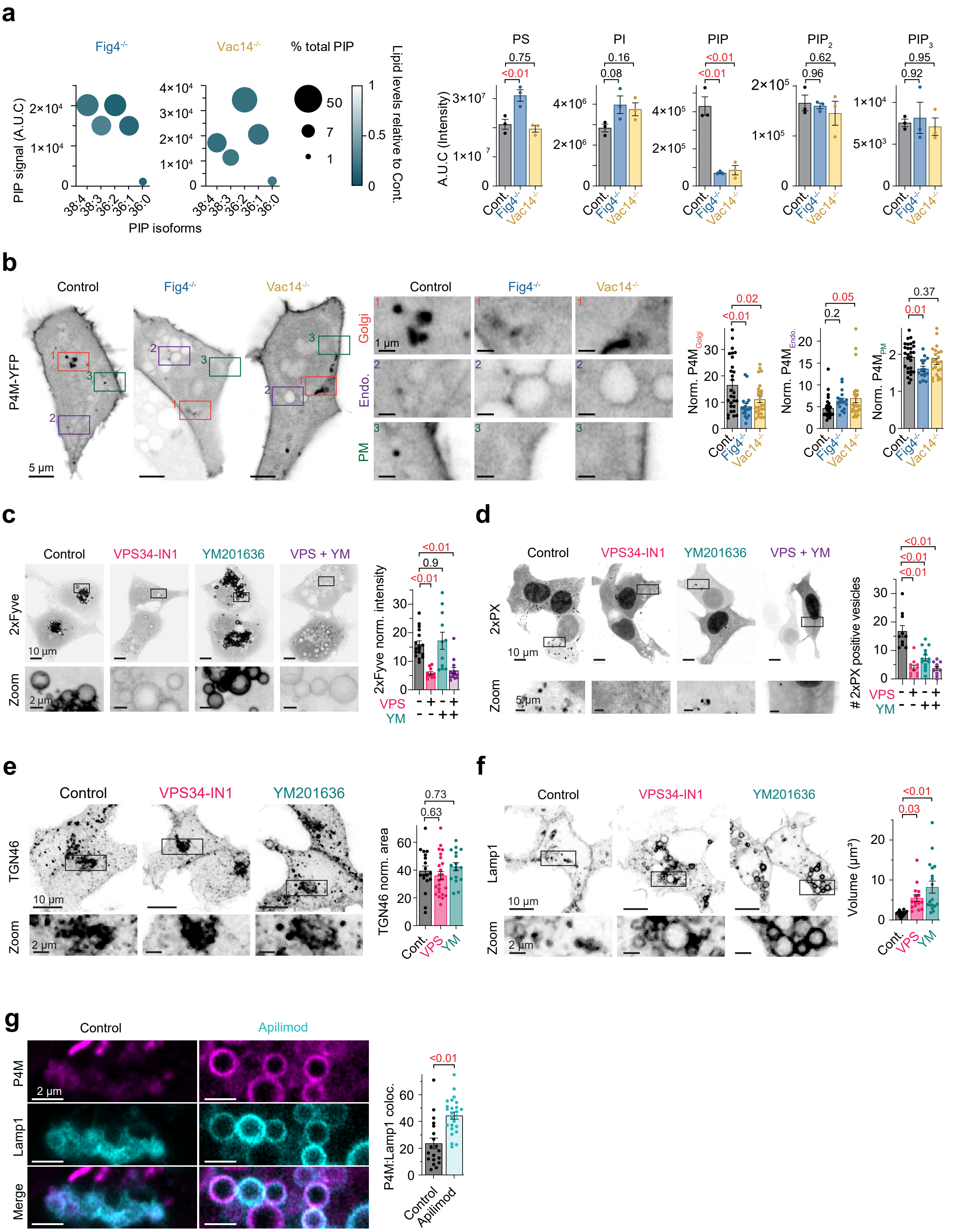

Supplementary Figure 1. Loss of PIKfyve, PI3K, Fig4, or Vac14 function alters cellular phosphoinositide species and lysosome volume. a, UPLC-MS/MS analysis of different PIP isoforms from Fig4$^{-/-}$ or VAC14$^{-/-}$ cells. **b,** Left: representative confocal images of P4M-YFP in control, Fig4- and Vac14-deficient cells. Right: quantification of P4M-YFP normalized intensity in Golgi, endo-lysosomal, and plasma membrane compartments. Control: n = 28 cells; Fig4$^{-/-}$: n = 18; Vac14$^{-/-}$: n = 25. **c,** Left: representative live AiryScan images from COS7 cells expressing the PI(3)P lipid biosensor GFP-2xFyve under control, VPS34-IN1, YM201636, or dual treatment conditions. Right: analysis of 2xFyve intensity from each condition. Control: n = 17 cells; VPS34-IN1: n = 11; YM201636: n = 11; and dual treatment: n = 13. **d,** Left: representative live AiryScan images from HEK293t cells expressing the PI(3,5)P_2 lipid biosensor GFP-2xPX under control, VPS34-IN1, YM201636, or dual treatment conditions. Right: analysis of GFP-2xPX positive vesicles from each condition. Control: n = 10 cells; VPS34-IN1: n = 11; YM201636: n = 13; and dual treatment: n = 10. **e,** Left: representative live AiryScan images from HEK293t cells expressing the Emerald-TGN46, under control, VPS34-IN1, YM201636, or dual treatment conditions. Right: analysis of Emerald-TGN46 area from each condition. Control: n = 18 cells; VPS34-IN1: n = 24; and YM201636: n = 17. **f,** Left: representative live AiryScan images from HEK293t cells expressing GFP-Lamp1 under control, VPS34-IN1, and YM201636 conditions. Right: analysis of the average GFP-Lamp1 volume from each condition. Control: n = 15 cells; VPS34-IN1: n = 15; and YM201636: n = 18. **g,** Left: representative confocal images of HEK293t cells expressing P4M-mCherry and mTagBFP2-Lysosomes-20 (Lamp1) in control (DMSO) or Apilimod-treated conditions. Right: quantification of P4M area within Lamp1 signal. Control: n = 19 cells; Apilimod: n = 25. For a-g, data are presented as mean ± s.e.m, and the statistical significance was determined using a one-way ANOVA (a-f) or a two-tailed Student's t-test (g).

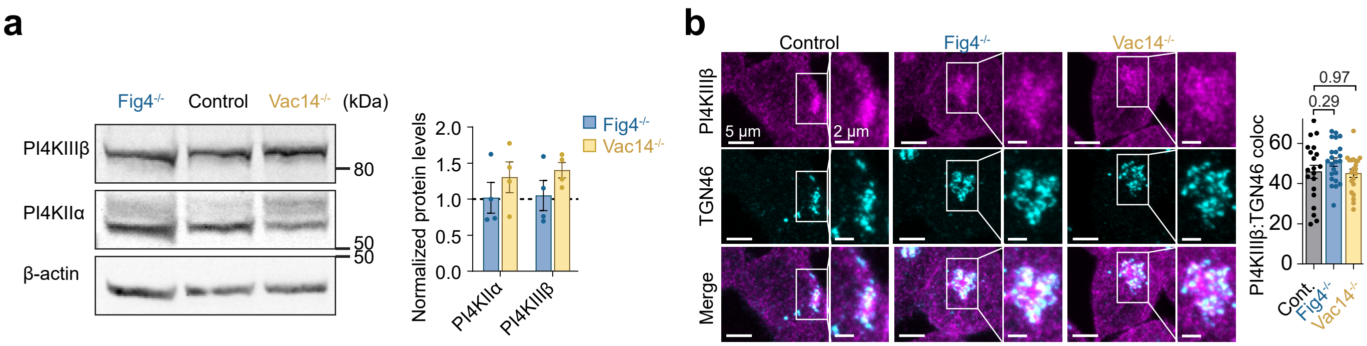

Supplementary Figure 2. Loss of Fig4 or Vac14 function does not alter PI4KIIIβ expression or distribution. a, Left: Western blot of PI4KIIIβ, PI4KIIα, and β-actin from Fig4$^{-/-}$, control, and Vac14$^{-/-}$ cells. Blots are representative of 4 independent replicates. Right: quantification of protein band intensity, normalized to β-actin signal. **b,** Left: representative AiryScan images from Control, Fig4$^{-/-}$, and Vac14$^{-/-}$ cells fixed and stained for PI4KIIIβ (top) and TGN46 (middle). Bottom row shows merged channels. Right: quantification of PI4KIIIβ:TGN46 colocalization. Control: n = 20 cells; Fig4$^{-/-}$: n = 23; Vac14$^{-/-}$: n = 21. For a-b, data are presented as mean ± s.e.m. For b, statistical significance was determined using a one-way ANOVA.

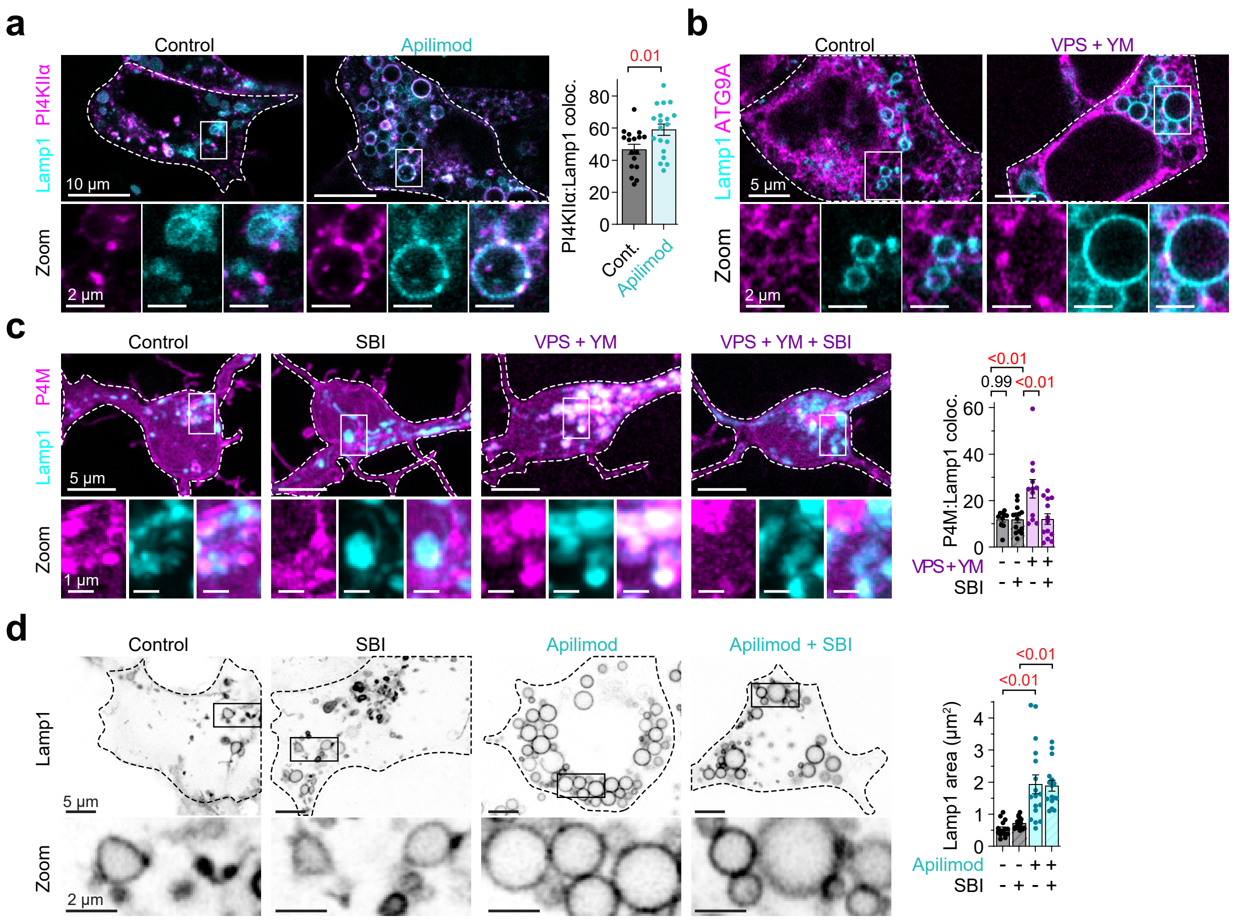

Supplementary Figure 3. PIKfyve complex deficiency drives ULK1-dependent trafficking of ATG9A to lysosomes. a, Left: representative live AiryScan images from HEK293t cells expressing PI4KIIα-GFP and mTagBFP2-Lysosomes-20 (Lamp1) under control or Apilimod-treated conditions. Right: quantification of PI4KIIα:Lamp1 colocalization. Control: n = 17 cells; Apilimod: n = 19. **b,** Left: representative live AiryScan images from HEK293t cells expressing ATG9A-RFP and Lamp1-GFP under control or VPS34-IN1 and YM201636 treatment conditions. Right: quantification of ATG9A:Lamp1 colocalization. Data is from the same dataset as presented in Fig. 4e. Control: n = 14 cells; VPS34-IN1 and YM201636: n = 17. **c,** Left: Confocal images of live cortical neurons expressing P4M-YFP and Lamp1-RFP, and treated with either DMSO (control) or VPS34-IN1 and YM201636, and pre-incubated with ULK-1 inhibitor SBI-0206965. Insets show enlarged views of P4M centered on lysosomal regions. Right: Quantification of P4M area within Lamp1 signal (%). Control: n = 10 neurons; SBI: n = 14; VPS34-IN1 + YM201636: n = 12; SBI + VPS34-IN1 + YM201636: n = 13. **d,** Left: Confocal images of live HEK293t cells expressing Lamp1, and treated with either DMSO (control), SBI, Apilimod, or Apilimod and SBI. Insets show enlarged views of Lamp1 signals. Right: Quantification of Lamp1 area in each condition. Control: n = 15 cells; SBI: n = 19; Apilimod: n = 17; Apilimod + SBI: n = 16. Data are presented as mean ± s.e.m and the statistical significance was determined using a two-tailed Student's t-test (a, b) or two-way ANOVA (c, d).

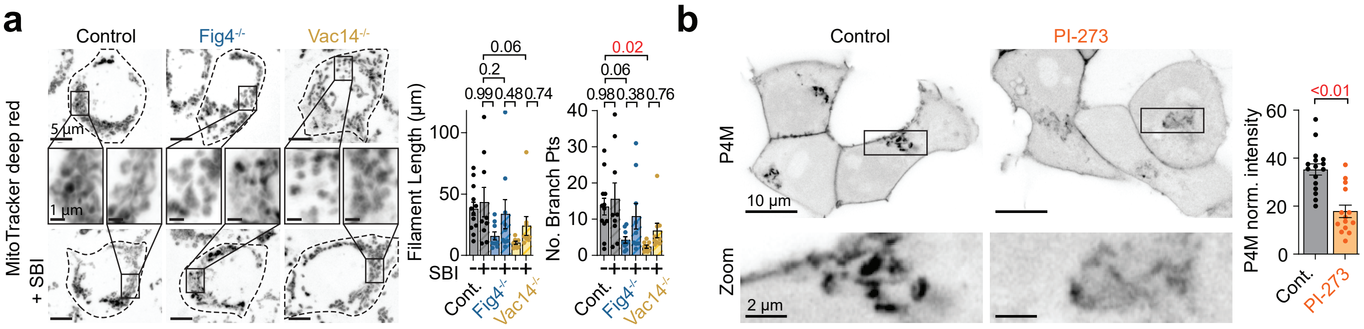

Supplementary Figure 4. Mitochondrial morphology is dependent upon PIKfyve complex function. a, Left: Confocal images of control, Fig4- and Vac14-deficient cells stained with MitoTracker Deep Red and pre-incubated with or without SBI-0206965. Control: n = 12 cells; Fig4$^{-/-}$: n = 9; Vac14$^{-/-}$: n = 9; Control + SBI: n = 9; Fig4$^{-/-}$ + SBI: n = 9; Vac14$^{-/-}$ + SBI: n = 9. Right: Quantification of the average mitochondrial filament length (µm) and number of branch points. **b,** Left: Confocal images of DMSO (control) and PI-273-treated HEK293t cells expressing P4M-YFP. Insets show enlarged views of P4M in the TGN region. Right: Quantification of average P4M-YFP fluorescence intensity relative to the cytoplasm in control (n = 16 cells) and PI-273-treated cells (n = 14). The statistical significance was determined using a 2-way ANOVA (a) or two-tailed Student's t-test (b). All data are presented as mean ± s.e.m.

Supplementary Table

Plasmids

P4M-YFP	Kruse *et al.* (2016)	N/A
mCherry-P4M	Hammond *et al.* (2014)	N/A
2xFyve-GFP	Addgene	RRID:Addgene_140047
GFP-2xPX	Addgene	RRID: Addgene_205130
NES-PHx3-GFP	Addgene	RRID:Addgene_ 116855
mCherry-TGN46	Addgene	RRID:Addgene_55145
Emerald-TGN46	Addgene	RRID:Addgene_54279
PI4KIIα-GFP	Balla *et al.* (2002)	N/A
Lamp1-mGFP	Addgene	RRID:Addgene_34831
Lamp1-RFP	Addgene	RRID:Addgene_1817
AIMTOR T757	Addgene	RRID:Addgene_140828
AIMTOR A757	Addgene	RRID:Addgene_140829
ATG9A-mCherry	Addgene	RRID:Addgene_197393
pLJM1-FLAG-GFP-OSBP	Addgene	RRID:Addgene_134659
ORP9-GFP	Li *et al.* (2024)	N/A
mCherry-Gal3	Addgene	RRID:Addgene_85662
Lact-C2-GFP	Addgene	RRID:Addgene_22852
LAMP1-pHluorin	Addgene	RRID:Addgene_171720
TagBFP2-C1-SACM1LdeltaTMD-Fis1tail	Addgene	RRID:Addgene_ 220077
TagBFP2-C1-SACM1LdeltaTMD-C389S-Fis1tail	Addgene	RRID:Addgene_ 220079
mTagBFP2-TOMM20-N-10	Addgene	RRID:Addgene_55328
iRFP-Sec61beta	Addgene	RRID:Addgene_108125
mTagBFP2-Lysosomes-20	Addgene	RRID:Addgene_55308

Oligonucleotide Primers

sgATG9A	Integrated DNA Technologies	Hs.Cas9.ATG9A.1.AA<br>Sequence:<br>CCTCGGCGACGTGCACCAAC

Antibodies

Mouse monoclonal PI4KIIα	Santa Cruz	Cat# sc-390026
Mouse monoclonal PI4KIIIβ	BD Biosciences	Cat# 611816; RRID: AB_399296
Rabbit polyclonal TGN46	NovusBio	Cat# NBP1-49643; RRID: AB_10011762
Mouse monoclonal β-actin	Thermo Fisher Scientific	Cat# MA1-91399, RRID: AB_2273656
Rabbit monoclonal ORP1L	Abcam	Cat# Ab131165 ; RRID:AB_11155305
Rabbit polyclonal DHHC3	Abcam	Cat# ab31837; RRID: AB_742236
Rabbit monoclonal Drp1	Abcam	Cat# ab184247; RRID:AB_2895215
Mouse monoclonal Lamp1	Abcam	Cat# ab25630; RRID: AB_470708
Rabbit polyclonal Lamp1	Thermo Fisher Scientific	Cat# PA1-654A; RRID:AB_2134611
Rabbit monoclonal mTOR	Cell Signaling Technology	Cat# 2983; RRID:AB_2105622
Rabbit polyclonal Phospho-ULK1	Cell Signaling Technology	Cat# 6888; RRID:AB_10829226
Rabbit monoclonal ULK1	Cell Signaling Technology	Cat# 8054; RRID:AB_11178668

Rabbit monoclonal Phospho-4E-BP1	Cell Signaling Technology	Cat# 2855; RRID:AB_560835
Rabbit monoclonal 4E-BP1	Cell Signaling Technology	Cat# 9644; RRID:AB_2097841
Rabbit polyclonal GAPDH	Proteintech	Cat# 10494-1-AP; RRID:AB_2263076
Goat anti-Rabbit, Alexa Fluor 647	Invitrogen	Cat# A-21245; RRID:AB_2535813
Goat anti-Rabbit, Alexa Fluor 488	Invitrogen	Cat# A-11034; RRID: AB_2576217
Goat anti-Mouse IgG1, Alexa Fluor 488	Invitrogen	Cat# A-21121; RRID:AB_2535764
Goat anti-Mouse IgG1, Alexa Fluor 647	Invitrogen	Cat# A-21240, RRID:AB_2535809
Goat anti-Mouse 800CW	LI-COR Biosciences	Cat# 925-32210, RRID: AB_2687825
Peroxidase IgG Fraction Monoclonal Mouse Anti-Rabbit IgG	Jackson Immunoresearch	Cat# 211-032-171; RRID: AB_2339149

Chemicals

B27	Gibco	Cat# 17504-044
Glutamax	Gibco	Cat# 35050-061
Apilimod	Millipore Sigma	Cat# SML2974
YM201636	ApexBio	Cat# B2189
VPS34-IN1	Selleckchem	Cat# S7980
SBI-0206965	MedChemExpress	Cat# HY-16966/CS-5016
PI-273	MedChemExpress	Cat# HY-103489
Torin1	Tocris	Cat# 4247
Lipofectamine 2000	Invitrogen	Cat# 11668-027
Lipofectamine LTX	Invitrogen	Cat# 15338-030
JetOPTIMUS	Polypus	Cat# 101000051
Paraformaldehyde	Electron Microscopy Sciences	Cat# 15710
cOmplete™, Mini Protease Inhibitor Cocktail	Roche	Cat# 11836153001
Nano-Glo Live Cell Assay System	Promega	Cat# N2011
Filipin	Sigma	Cat# F9765
LLOME	Millipore Sigma	Cat# L7393
MitoTracker Deep Red	Invitrogen	Cat# M22426
Seahorse XF Cell Mito Stress Kit	Agilent	Cat# 103015-100
SEA BLOCK Blocking Buffer	Thermo Scientific	Cat# 37527
Complete Mini protease inhibitor cocktail	Roche	Cat# 11836170001
OSW-1	Cayman Chemical	Cat# 30310
