# Supplementary material for: Disruption of the PIKfyve complex unveils an adaptive mechanism to promote lysosomal repair and mitochondrial homeostasis
Source: Nat Commun. 2025 Nov 28;16:10761. doi: 10.1038/s41467-025-65798-6 (PMC12663346; doi:10.1038/s41467-025-65798-6)
Supplement: Supplementary file 2 — Reporting Summary [file 41467_2025_65798_MOESM2_ESM.pdf]

nature portfolio

Corresponding author(s): Eamonn Dickson

Last updated by author(s): Oct 22, 2025

Reporting Summary

Nature Portfolio wishes to improve the reproducibility of the work that we publish. This form provides structure for consistency and transparency in reporting. For further information on Nature Portfolio policies, see our Editorial Policies and the Editorial Policy Checklist.

Statistics

For all statistical analyses, confirm that the following items are present in the figure legend, table legend, main text, or Methods section.

n/a	Confirmed	
☐	☒	The exact sample size (n) for each experimental group/condition, given as a discrete number and unit of measurement
☐	☒	A statement on whether measurements were taken from distinct samples or whether the same sample was measured repeatedly
☐	☒	The statistical test(s) used AND whether they are one- or two-sided<br>*Only common tests should be described solely by name; describe more complex techniques in the Methods section.*
☐	☒	A description of all covariates tested
☐	☒	A description of any assumptions or corrections, such as tests of normality and adjustment for multiple comparisons
☐	☒	A full description of the statistical parameters including central tendency (e.g. means) or other basic estimates (e.g. regression coefficient) AND variation (e.g. standard deviation) or associated estimates of uncertainty (e.g. confidence intervals)
☒	☐	For null hypothesis testing, the test statistic (e.g. F, t, r) with confidence intervals, effect sizes, degrees of freedom and P value noted<br>*Give P values as exact values whenever suitable.*
☒	☐	For Bayesian analysis, information on the choice of priors and Markov chain Monte Carlo settings
☒	☐	For hierarchical and complex designs, identification of the appropriate level for tests and full reporting of outcomes
☒	☐	Estimates of effect sizes (e.g. Cohen's d, Pearson's r), indicating how they were calculated

Our web collection on statistics for biologists contains articles on many of the points above.

Software and code

Policy information about availability of computer code

Data collection	Imaging data was collected using the following softwares: Zeiss Zen (Zeiss Zen v2.3 SP1), and Micromanager (1.4.21). Biochemistry data was imaged using a Azure Biosystems Sapphire Biomolecular Imager. Mitochondrial oxygen consumption rate was measured using a Seahorse XFe96 Extracellular Flux Analyzer. Transmission Electron Microscopy images were acquired using Velox Acquisition software.
Data analysis	Image analysis was performed using the following softwares: FIJI/ImageJ (v. 2.14.0/1.54f) and Imaris x64 10.1.1. Quanification of protein expression on western blots was performed using FIJI/ImageJ (v. 2.14.0/1.54f). Mitochondrial oxygen consumption rate data were analyzed using the Agilent Seahorse Wave Desktop (v. 2.6.4.24) and Microsoft Excel (v. 2408 Build 16.0.17928.20114) softwares. For lipid mass spectometry, elution profiles were quantified by integrating the area under peaks using MassLynx software.

For manuscripts utilizing custom algorithms or software that are central to the research but not yet described in published literature, software must be made available to editors and reviewers. We strongly encourage code deposition in a community repository (e.g. GitHub). See the Nature Portfolio guidelines for submitting code & software for further information.

Data

Policy information about availability of data

All manuscripts must include a data availability statement. This statement should provide the following information, where applicable:

- Accession codes, unique identifiers, or web links for publicly available datasets
- A description of any restrictions on data availability
- For clinical datasets or third party data, please ensure that the statement adheres to our policy

The data supporting the findings of this work can be found in the source data file. The lipidomic data generated in this study have been deposited in the MetaboLights database under accession code: REQ20251021214068.

Research involving human participants, their data, or biological material

Policy information about studies with human participants or human data. See also policy information about sex, gender (identity/presentation), and sexual orientation and race, ethnicity and racism.

Reporting on sex and gender	N/A
Reporting on race, ethnicity, or other socially relevant groupings	N/A
Population characteristics	N/A
Recruitment	N/A
Ethics oversight	N/A

Note that full information on the approval of the study protocol must also be provided in the manuscript.

Field-specific reporting

Please select the one below that is the best fit for your research. If you are not sure, read the appropriate sections before making your selection.

☒ Life sciences ☐ Behavioural & social sciences ☐ Ecological, evolutionary & environmental sciences

For a reference copy of the document with all sections, see nature.com/documents/nr-reporting-summary-flat.pdf

Life sciences study design

All studies must disclose on these points even when the disclosure is negative.

Sample size	The sample sizes for our biochemical and imaging analyses were chosen based on previous published literature with similar experimental design and our previous published works.
Data exclusions	Data outliers were determined using a two-sided Grubbs' test and subsequently excluded from the analyses.
Replication	To account for variability in sample preparation, animals, ambient conditions, etc, findings are indicated in the data source file.
Randomization	Samples were randomly allocated into experimental treatment groups.
Blinding	The investigators were not blinded to experimental groups during data acquisition as none of the analyses were based on subjective observations.

Reporting for specific materials, systems and methods

We require information from authors about some types of materials, experimental systems and methods used in many studies. Here, indicate whether each material, system or method listed is relevant to your study. If you are not sure if a list item applies to your research, read the appropriate section before selecting a response.

Materials & experimental systems

n/a	Involved in the study
	☒ Antibodies
	☒ Eukaryotic cell lines
☒	Palaeontology and archaeology
	☒ Animals and other organisms
☒	Clinical data
☒	Dual use research of concern
☒	Plants

Methods

n/a	Involved in the study
☒	ChIP-seq
☒	Flow cytometry
☒	MRI-based neuroimaging

Antibodies

Antibodies used

Mouse monoclonal PI4KIIα (Santa Cruz, Cat# sc-390026); Mouse monoclonal PI4KIIIβ (BD Biosciences, Cat# 611816; RRID: AB_399296); Rabbit polyclonal TGN46 (NovusBio, Cat# NBP1-49643; RRID:AB_10011762); Mouse monoclonal β-actin (Thermo Fisher Scientific, Cat# MA1-91399, RRID: AB_2273656); Rabbit polyclonal DHHC3 (Abcam, Cat# ab31837; RRID: AB_742236); Rabbit monoclonal Drp1 (Abcam, Cat# ab184247; RRID:AB_2895215); Mouse monoclonal Lamp1 (Abcam, Cat# ab25630; RRID: AB_470708); Rabbit polyclonal Lamp1 (Thermo Fisher Scientific, Cat# PA1-654A; RRID:AB_2134611); Rabbit monoclonal mTOR (Cell Signaling Technology, Cat# 2983, RRID:AB_2105622); Goat anti-Rabbit, Alexa Fluor 647 (Invitrogen, Cat# A-21245; RRID:AB_2535813); Goat anti-Rabbit, Alexa Fluor 488 (Invitrogen, Cat# A-11034; RRID: AB_2576217); Goat anti-Mouse IgG1, Alexa Fluor 488 (Invitrogen, Cat# A-21121; RRID:AB_2535764); Goat anti-Mouse IgG1, Alexa Fluor 647 (Invitrogen, Cat# A-21240, RRID:AB_2535809); Goat anti-Mouse 800CW (LI-COR Biosciences, Cat# 925-32210, RRID: AB_2687825).

Validation

All antibodies are commercially available or upon request by source, and the applications have been tested by the manufacturer and by us in previous studies:

Mouse monoclonal PI4KIIα (Santa Cruz, Cat# sc-390026; IF: 1:50). Data sheet: https://datasheets.scbt.com/sc-390026.pdf; Citation: Kutchukian, C. et al. NPC1 regulates the distribution of phosphatidylinositol 4-kinases at Golgi and lysosomal membranes. The EMBO Journal 40, e105990 (2021).

Mouse monoclonal PI4KIIIβ (BD Biosciences, Cat# 611816; WB: 1:1000). Data sheet: https://www.bdbiosciences.com/content/dam/bdb/products/global/reagents/microscopy-imaging-reagents/immunofluorescence-reagents/611xxx/6118xx/611817_base/pdf/611816.pdf

Rabbit polyclonal TGN46 (NovusBio, Cat# NBP1-49643; IF: 1:300). Data sheet: https://www.novusbio.com/PDFs/NBP1-49643.pdf; Citation: Kutchukian, C. et al. NPC1 regulates the distribution of phosphatidylinositol 4-kinases at Golgi and lysosomal membranes. The EMBO Journal 40, e105990 (2021).

Rabbit polyclonal DHHC3 (Abcam, Cat# ab31837; IF: 1:50). Data sheet: https://doc.abcam.com/datasheets/active/ab31837/en-us/godz-dhhc-3-antibody-ab31837.pdf; Citation: Kutchukian, C. et al. NPC1 regulates the distribution of phosphatidylinositol 4-kinases at Golgi and lysosomal membranes. The EMBO Journal 40, e105990 (2021).

Rabbit monoclonal Drp1 (Abcam, Cat# ab184247; IF: 1:250). Data sheet: https://doc.abcam.com/datasheets/active/ab184247/en-us/drp1-antibody-epr19274-ab184247.pdf

Mouse monoclonal Lamp1 (Abcam, Cat# ab25630; IF: 1:10). Data sheet: https://doc.abcam.com/datasheets/active/ab25630/en-us/lamp1-antibody-h4a3-ab25630.pdf; Citation: Kutchukian, C. et al. NPC1 regulates the distribution of phosphatidylinositol 4-kinases at Golgi and lysosomal membranes. The EMBO Journal 40, e105990 (2021).

Rabbit polyclonal Lamp1 (Thermo Fisher Scientific, Cat# PA1-654A; IF: 1:100). Data sheet: https://www.thermofisher.com/order/genome-database/dataSheetPdf?producttype=antibody&productsubtype=antibody_primary&productId=PA1-654A&version=Local

Rabbit monoclonal mTOR (Cell Signaling Technology, Cat# 2983; IF: 1:200). Data sheet: https://www.cellsignal.com/products/primary-antibodies/mtor-7c10-rabbit-mab/2983; Citation: Kutchukian, C. et al. NPC1 regulates the distribution of phosphatidylinositol 4-kinases at Golgi and lysosomal membranes. The EMBO Journal 40, e105990 (2021).

Goat anti-Rabbit, Alexa Fluor 647 (Invitrogen, Cat# A-21245; IF: 1:1000). Data sheet: https://www.thermofisher.com/order/genome-database/dataSheetPdf?producttype=antibody&productsubtype=antibody_secondary&productId=A-21245&version=Local. Citation: Horvath, J.D. et al. α-Synuclein-dependent increases in PIP5K1γ drive inositol signaling to promote neurotoxicity. Cell Reports 42, 113244 (2023).

Goat anti-Rabbit, Alexa Fluor 488 (Invitrogen, Cat# A-11034; IF: 1:1000). Data sheet: https://www.thermofisher.com/order/genome-database/dataSheetPdf?producttype=antibody&productsubtype=antibody_secondary&productId=A-11034&version=Local; Citation: Kutchukian, C. et al. NPC1 regulates the distribution of phosphatidylinositol 4-kinases at Golgi and lysosomal membranes. The EMBO Journal 40, e105990 (2021).

Goat anti-Mouse IgG1, Alexa Fluor 488 (Invitrogen, Cat# A-21121; IF: 1:1000). Data sheet: https://www.thermofisher.com/antibody/product/Goat-anti-Mouse-IgG1-Cross-Adsorbed-Secondary-Antibody-Polyclonal/A-21121.

Goat anti-Mouse IgG1, Alexa Fluor 647 (Invitrogen, Cat# A-21240; IF: 1:1000). Data sheet: https://www.thermofisher.com/antibody/product/Goat-anti-Mouse-IgG1-Cross-Adsorbed-Secondary-Antibody-Polyclonal/A-21240.

Goat anti-Mouse 800CW (LI-COR Biosciences, Cat# 925-32210). Data sheet: https://www.licor.com/bio/reagents/irdye-800cw-goat-anti-mouse-igg-secondary-antibody; Citation: Kutchukian, C. et al. NPC1 regulates the distribution of phosphatidylinositol 4-kinases at Golgi and lysosomal membranes. The EMBO Journal 40, e105990 (2021).

Eukaryotic cell lines

Policy information about cell lines and Sex and Gender in Research

Cell line source(s)	Fig4 knock-out (Fig4-/-) and Vac14 knock-out (Vac14-/-) HAP1 cells were a gift from Dr. J.O. Kitzman (University of Michigan, Ann Arbor, MI).<br>HEK293t cells were purchased from Sigma, Cat #96121229<br>HEK293 stably expressing Cas9 (HEK293 Cas9, were purchased from ATCC, Cat #CRL-1573Cas9<br>COS7 cells were kindly gifted from Dr. J. Nunnari, University of California, Davis, CA.
Authentication	Cell lines from Sigma were authenticated by STR profiling. Cell lines obtained from other sources were authenticated by performing WB or fluorescence imaging.
Mycoplasma contamination	Cell lines were regularly tested for mycoplasm contamination and were not contaminated.
Commonly misidentified lines<br>(See ICLAC register)	No commonly misidentified cell lines were used in this study.

Animals and other research organisms

Policy information about studies involving animals; ARRIVE guidelines recommended for reporting animal research, and Sex and Gender in Research

Laboratory animals	Embryonic day 18 C57/B6 Male and Female animals were used to generate neuronal cultures.
Wild animals	The study did not involve wild animals.
Reporting on sex	Neuronal isolations were performed on embryonic day 18 male and female pups and samples pooled.
Field-collected samples	The study did not involve any field-collected samples.
Ethics oversight	Animals studies were approved and overseen by the University of California Davis Animal Care and Use Committee (protocol #: 20974).

Note that full information on the approval of the study protocol must also be provided in the manuscript.

Plants

Seed stocks	N/A
Novel plant genotypes	N/A
Authentication	N/A
